# Delphinidin Ameliorates Hepatic Triglyceride Accumulation in Human HepG2 Cells, but Not in Diet-Induced Obese Mice

**DOI:** 10.3390/nu10081060

**Published:** 2018-08-10

**Authors:** Marcela Parra-Vargas, Ana Sandoval-Rodriguez, Roberto Rodriguez-Echevarria, Jose Alfredo Dominguez-Rosales, Arturo Santos-Garcia, Juan Armendariz-Borunda

**Affiliations:** 1 Institute for Molecular Biology in Medicine and Gene Therapy, Department of Molecular Biology and Genomics, CUCS, University of Guadalajara, Guadalajara 44340, Jalisco, Mexico; marcela.parrav@gmail.com (M.P.-V.); anasol44@hotmail.com (A.S.-R.); roberto14n@gmail.com (R.R.-E.); 2 Chronic-Degenerative Diseases Institute, Department of Molecular Biology and Genomics, CUCS, University of Guadalajara, Guadalajara 44340, Jalisco, Mexico; dominque14@yahoo.com; 3 Tecnologico de Monterrey, Campus Guadalajara, Guadalajara 45138, Jalisco, Mexico; arturo.santos@itesm.mx

**Keywords:** anthocyanins, antioxidants, delphinidin, non-alcoholic fatty liver disease, obesity, Western-style diet

## Abstract

Anthocyanin consumption is linked to benefits in obesity-related metabolic alterations and non-alcoholic fatty liver disease (NAFLD), though the functional role of delphinidin (Dp) is yet to be established. Therefore, this study examined the effects of Dp on metabolic alterations associated with NAFLD, and molecular mechanisms in HepG2 cells and diet-induced obese mice. Cells incubated with palmitate to induce lipid accumulation, concomitantly treated with Dp, reduced triglyceride accumulation by ~53%, and downregulated gene expression of *CPT1A*, *SREBF1*, and *FASN* without modifying AMP-activated protein kinase (AMPK) levels. C57BL/6Nhsd mice were fed a standard diet (control) or a high-fat/high-carbohydrate diet (HFHC) for 16 weeks. Mice in the HFHC group were subdivided and treated with Dp (HFHC-Dp, 15 mg/kg body weight/day) or a vehicle for four weeks. Dp did not affect body weight, energy intake, hyperglycemia, insulin resistance, or histological abnormalities elicited by the HFHC diet. Furthermore, the messenger RNA (mRNA) expressions of *Acaca*, and *Fasn* in hepatic or epididymal adipose tissue, and the hepatic sirtuin 1 (SIRT1)/liver kinase B1 (LKB1)/AMPK and proliferator-activated receptor alpha (PPARα) signaling axis did not significantly change due to the HFHC diet or Dp. In summary, Dp effectively reduced triglyceride accumulation in vitro through the modulation of lipid metabolic gene expression. However, a dose of Dp administrated in mice simulating the total daily anthocyanin intake in humans had no effect on either metabolic alterations or histological abnormalities associated with HFHC diets.

## 1. Introduction

Non-alcoholic fatty liver disease (NAFLD) is characterized by an excess of hepatic lipids as a consequence of a metabolic imbalance between the input/output of fatty acids, mostly associated with obesity [1]. The worldwide prevalence of NAFLD parallels the current obesity context [2]. Furthermore, it is now the most prevalent chronic liver disorder in Western countries [3]. Histologically, NAFLD encompasses a spectrum of morphological changes ranging from simple steatosis (SS) characterized by steatosis alone, to more progressive non-alcoholic steatohepatitis (NASH) with the presence of inflammation and hepatocyte ballooning [4]. The progression of SS to NASH is more likely when obesity and insulin resistance are present [5]. The persistence of hepatic inflammation, which is predominant in NASH, is strongly associated with fibrosis, cirrhosis, and, in some cases, hepatocarcinoma [4,6,7]. Despite the development of a remarkable understanding of the possible molecular targets involved in pathogenesis in the last few years, current pharmacological treatments for NAFLD are still limited. First-line treatment is based on body weight management, mainly aimed at reducing stored fat through modifying lifestyle factors such as dietary habits [8]. In addition to modulating caloric intake, dietary recommendations include the consumption of naturally occurring bioactive compounds with antioxidant and biological properties which may have an essential role as adjuvants in the treatment [9].

Anthocyanins are natural flavonoids that belong to a polyphenol group with known potent antioxidant activity [10,11]. They are water-soluble pigments widely present in food plants that are part of the human diet, of which regular intake is associated with health benefits [12,13]. Structurally, anthocyanins are the glycosylated forms of anthocyanidins, and one of the major anthocyanidins found in nature is delphinidin (Dp) [13]. Among anthocyanidins, Dp shows higher radical scavenging activity due to the presence of a large number of hydroxyl groups in its structure which enhance reactivity [14]. Dp-based anthocyanins are predominantly in bluish-to-black pigmented foods such as blueberry, black currant, black bean, Concord grape, maqui berry, and *Hibiscus sabdariffa* [11,15,16,17,18]. In addition to their antioxidants properties, it was reported in a number of clinical trials, as well as in vitro and in vivo studies, that anthocyanins could play an important role in modulating metabolism, thus protecting against NAFLD-related metabolic alterations [19,20,21,22]. Purified mulberry anthocyanins, mainly composed of cyanidin-based anthocyanins, inhibited weight gain and reduced hepatic lipid content in diet-induced obese mice [23]. Likewise, a cyanidin-3-glucoside dietary supplementation for 12 weeks counteracted high-fat diet (HFD) effects, in which hepatic triglycerides were lower and hyperglycemia was ameliorated [24]. Nevertheless, a recent study reported different metabolic effects according to the anthocyanin profile of diverse berry powders. In diet-induced obese mice, an anthocyanin supplementation for 12 weeks from blackcurrant and blueberry improved insulin sensitivity, while those from maqui berry, Concord grape, blackberry, and black raspberry did not. Moreover, body weight decreased by 6% only with blackcurrant and Concord grape [17]. Based on these findings, a better understanding of the effects of anthocyanins and how they work at the molecular level is fundamental.

Continuously growing evidence suggests that metabolic improvements due to anthocyanins are through the activation of AMP-activated protein kinase (AMPK) [22,25,26], which inhibits de novo lipogenesis via the direct phosphorylation of the sterol response element-binding protein-1c (SREBP1c), thereby interfering its transcriptional activity on lipogenic rate-limiting enzymes such as fatty acid synthase (FAS) and acetyl-CoA carboxylase 1 (ACC1) [27]. Additionally, AMPK promotes fatty-acid oxidation by directly suppressing ACC1 activity, reducing malonil-CoA, which is an allosteric inhibitor of carnitine palmitoyltransferase A (CPT1A), a key regulator of fatty-acid oxidation [28]. The major lipid oxidation regulator in liver, peroxisome proliferator-activated receptor alpha (PPARα) is also stimulated by anthocyanins. In fact, cyanidin acts as an agonist ligand of PPARα, and reduces hepatic lipid concentrations [26,29]. Collectively, previously mentioned findings suggest that these molecular mechanisms support the notion of beneficial effects of anthocyanins in NAFLD.

The upstream AMPK kinase activator, liver kinase B1 (LKB1), directly phosphorylates (Thr 172) and activates AMPK. The activation of sirtuin 1 (SIRT1) by other polyphenols promoted AMPK activation in an LKB1-dependent mechanism [30]. Also, it was reported that SIRT1 regulates fat mobilization by increasing adipose triglyceride lipase (ATGL) [31], while hepatic ATGL overexpression promotes fatty-acid oxidation [32]. To our best knowledge, the SIRT1/LKB1/AMPK and PPARα signaling axis is yet to be revealed under the action of anthocyanins, and the precise functional role of Dp in NAFLD is yet to be established. Therefore, the aim of this study was to examine the effects of Dp on metabolic alterations associated with NAFLD, as well as the molecular mechanisms in a fatty liver cell model and an obesity-related NAFLD model in mice.

## 2. Materials and Methods

### 2.1. Chemicals

Dp and compound C (CC) were purchased from Cayman Chemical (Ann Arbor, MI, USA). Sodium palmitate (PA) and non-esterified fatty acid-free bovine serum albumin (NEFA-free BSA) were obtained from Sigma-Aldrich (St. Louis, MO, USA). Antibodies for phosphorylated AMPK (p-AMPKα Thr-172) and LKB1 were purchased from Cell Signaling Technology (Beverly, MA, USA), and antibodies for AMPKα 1/2, SIRT1, PPARα, and β-actin were obtained from Abcam (Cambridge, MA, USA). All other chemicals used were of reagent grade.

### 2.2. Cell Culture

HepG2 cells were donated by the National Institute of Genomic Medicine (INMEGEN, Mexico City, Mexico), cultured in Dulbecco’s modified Eagle’s medium (DMEM; Corning Inc., Oneonta, NY, USA), supplemented with 10% fetal bovine serum (FBS; Biowest, Riverside, MO, USA) and an antibiotic/antimycotic mixture at 1× (Antibiotic/Antimycotic 100×, Gibco, Grand Island, NY, USA) at 37 °C in a humidified atmosphere of 95% air and 4.5% CO_2_. After reaching 80% confluence, cells were subcultured in six-well plates at 6.5 × 10^4^ in each well for different experiments. Prior to performing assays, cells were serum-starved for 24 h.

### 2.3. Preparation of PA and Intracellular Lipid Accumulation

PA was used to generate intracellular lipid accumulation. Firstly, PA was conjugated with NEFA-free BSA. A 100 mM PA stock solution was prepared by dissolving 27.8 mg of PA in 1 mL of sterile water by alternating vortexing and heating at 70 °C. Briefly, a 5 mM PA solution was prepared by adding 200 μL from the previous PA solution to 3.8 mL of serum-free DMEM with 5% NEFA-free BSA. Later, 5 mM PA solution was shaken at 140 rpm at 40 °C for 1 h. This solution was immediately used to treat the cells. Cells were exposed to different PA concentrations (0.5 mM and 1 mM) contained in medium for 24 h. Cells treated with 5% NEFA-free BSA were considered as the control. Based on the results (Figure S1), the fatty liver cell model was established by incubating HepG2 with 1 mM PA concentration.

### 2.4. Dp Treatment on the Fatty Liver Cell Model

A Dp stock solution at 88.58 mM was prepared by dissolving 30 mg of Dp in 1 mL of dimethyl sulfoxide (DMSO). Consequently, to establish an experimental Dp dose (100 μM or 180 μM) [33], HepG2 cells were treated concomitantly with PA for 24 h to evaluate cell viability and triglyceride accumulation. Afterward, cells were incubated with both Dp concentrations for 1 h without PA, and AMPK phosphorylation levels were measured.

To evaluate the effects of Dp on the fatty liver cell model, HepG2 cells were treated with 180 μM Dp and PA for 24 h. Total lipid accumulation was visualized, and triglyceride levels were evaluated. Moreover, HepG2 cells were simultaneously incubated with PA, Dp, and CC (20 μM, an AMPK inhibitor) for 24 h in order to determine the role of AMPK in the Dp effect on triglyceride accumulation. Finally, to evaluate the gene expression of genes involved in lipid metabolism (*CPT1A*, *SREBF1*, *FASN*, and *PNPLA2*; Table S1) and the effect on AMPK phosphorylation levels, cells were pre-treated with PA for 24 h, and were then exposed to 180 μM Dp for one additional hour. In all abovementioned experiments, cells incubated with DMEM or DMSO + NEFA-free BSA were considered as controls. Other than the DMEM-incubated cells, culture media contained DMSO at 0.363%.

### 2.5. Animal Experiments

This research protocol was approved by the CUCS Research Committee from the University of Guadalajara (permission number: C.I. 005-2017). Four-week-old male C57BL/6Nhsd mice weighing 20–25 g were purchased from Envigo (Mexico City, Mexico). Mice were maintained in polycarbonate cages in a room at 22 ± 1 °C and an alternating 12-h light/dark cycle. After a week, mice were randomly allocated to two main groups and fed ad libitum with a standard diet and water (control; *n* = 6) or a high-fat diet and high-carbohydrate drinking water (45% kcal from fat, HFHC; *n* = 12) for 16 weeks to induce NAFLD. Subsequently, mice in the HFHC group were divided into subgroups (*n* = 6): one was treated orally once per day with Dp (15 mg/kg body weight (bw); HFHC-Dp), and the rest of the HFHC group received a vehicle (DMSO, final concentration 4.7%) for four weeks. All groups received an equal concentration of DMSO, and the total volume administered by oral gavage was 350 μL. At the end of the experimental period, mice were fasted for 4 h, and euthanized with tiletamine/zolazepam (15 mg/kg bw). Livers were weighed, and the tissue of the three main lobules was fixed with paraformaldehyde for histological examination. Samples of liver and epididymal adipose tissue were immediately collected, and stored at −80°C for further molecular experiments.

### 2.6. Diets, Food Intake, and Energy Intake

The control group with a standard diet (Prolab RMH 2500 5P14*) was provided with 3.04 kcal/g of energy (12%, 29%, and 59% kcal from fat, proteins, and carbohydrates, respectively), whereas the HFHC group (TestDiet 58V8) was supplied with 4.60 kcal/g (45%, 20%, and 35% kcal from fat, proteins, and carbohydrates, respectively) plus a high-carbohydrate drinking water at a concentration of 42 g/L (at a ratio of 55% fructose and 45% sucrose; 0.168 kcal/mL). Food intake was measured three times per week at 10–11 a.m., and energy ingestion was calculated from the consumed food and beverage. Ingestion-related data were analyzed in both phases: prior to treatment and during Dp treatment.

### 2.7. Cell Viability Assay

Cell viability was determined using a 3-(4,5-dimethylthiazol-2-yl)-2,5-diphenyltetrazolium bromide (MTT) cell proliferation assay kit (Cayman Chemical, Ann Arbor, MI, USA). HepG2 cells were seeded at 1 × 10^4^ per well into a 96-well plate with 100 μL of 10% FBS/DMEM for 24 h. Afterward, the medium was replaced by serum-free DMEM for 24 h. Then, cells were concomitantly incubated with Dp (100 μM or 180 μM) and PA for another 24 h. A volume of 10 μL of MTT was added to each well, before it was softly shaken, and incubated for 4 h. Microplates were then centrifuged at 1800 rpm for 5 min, and MTT-containing medium was removed. Finally, formazan crystals were dissolved with 150 μL of DMSO using soft agitation. Absorbance was measured at 570 nm with a spectrophotometer (Quant Microplate Spectrophotometer, Bio-Tek Instruments Inc., Winooski, VT, USA). The viability of the control (DMSO + NEFA-free BSA treated cells) was taken as 100% viable.

### 2.8. Determination of Intracellular Lipid Accumulation Levels

Neutral lipid accumulation (i.e., triglycerides, diacylglycerol, and cholesterol esters) was visualized by Oil Red O staining (ORO; Sigma-Aldrich, St. Louis, MO, USA). Firstly, cells were fixed with a 10% paraformaldehyde solution. Afterward, ORO working solution was added for 5 min, and washed with saline solution. Hematoxylin staining was used to identify cell nuclei. Representative microscopic images were captured using a 32× objective lens (Axio Vert.A1; Carl Zeiss AG Corporate Headquarters, Oberkochen, BW, Germany). Intracellular triglyceride levels were determined with an enzymatic kit, and performed according to the manufacturer’s instructions (Triglyceride Colorimetric Assay Kit; Cayman Chemical, Ann Arbor, MI, USA).

### 2.9. Weight, Fasting Glucose, and Insulin Sensitivity

The weight and fasting glucose levels in mice were recorded weekly during the study. Glucose levels were measured with a blood glucose meter (One Touch Ultra, LifeScan Inc., Wayne, PA, USA) in tail-vein blood after 4 h of food deprivation. At the end of the treatment, mice were fasted for 4 h to analyze the insulin tolerance test (ITT) after an intraperitoneal injection of human insulin (Humulin R; Eli Lilly, Indianapolis, IN, USA) at a standardized dose of 0.025 U contained in saline solution. Glucose levels were measure at 0, 30, 60, and 120 min after insulin injection. The area under the curve (AUC) was statistically analyzed.

### 2.10. Liver Histology Analysis

Small pieces of liver were fixed in a solution (4% paraformaldehyde, 0.1 M phosphate-buffered saline (PBS)) by immersion and embedded in paraffin. Subsequently, liver sections (5 μM) were stained with hematoxylin and eosin (H&E) and Masson’s trichrome stain. Evaluation of liver damage was based on the NAFLD activity score (NAS). The NAS represents the sum of the separate scores of NAS components: steatosis, lobular inflammation, and ballooning. Steatosis, lobular inflammation, and portal inflammation were scored 0–3, whereas ballooning was graded 0–2. Fibrosis was staged from 0–4. Images were captured using a camera (Infinity1, Olympus, Tokyo, Japan) at 20× magnification. A pathologist blindly performed the histological analysis.

### 2.11. Western Blotting Analysis

Aliquots (30 μg) of total protein extracted from cell lysates and liver tissue were loaded onto a 10% SDS-PAGE machine under reducing conditions, and were then transferred onto polyvinylidene difluoride (PVDF) membranes (Bio-Rad Laboratories, Inc., Hercules, CA, USA), which were blocked with 5% BSA in Tris-buffered saline containing 0.1% Tween 20 at room temperature for 1 h. Subsequently, membranes were immunoblotted with specific antibodies (p-AMPKα Thr172, 1:500; LKB1, AMPKα 1/2, SIRT1, PPARα and β-actin) overnight at 4 °C. Afterward, membranes were incubated with a peroxidase-conjugated secondary antibody (1:16,000) for 1 h at room temperature. Bands were detected using the BM Chemiluminescence Western Blotting Kit (Roche Applied Science, Penzberg, BY, Germany), and visualized through ChemiDoc XRS+ (Bio-Rad Laboratories, Inc., Hercules, CA, USA). Densitometry analysis was performed using the ImageStudio Lite program (LI-COR Biosciences, Lincoln, NE, USA). All band-density quantifications were normalized with β-actin.

### 2.12. Quantitative Real-Time PCR and Gene Expression Analysis

Total RNA from HepG2 cells, liver, and epididymal adipose tissue was extracted with Trizol reagent (Invitrogen, Carlsbad, CA, USA) according to the manufacturer’s instructions. RNA quantity and quality were measured with a NanoDrop spectrophotometer (Thermo Fisher Scientific, Waltham, MA, USA). Total RNA (2 μg) was reverse-transcribed to complementary DNA (cDNA) in a 50-μL final volume using 690-ng random primers, 0.72 mM deoxynucleotide triphosphate (dNTP) mix, 1× first-strand buffer, 3.6 mM dithiothreitol (DTT), 5 U of RNAase inhibitor, and 260 U Moloney Murine Leukemia Virus (M-MLV) reverse transcriptase (Invitrogen, Carlsbad, CA, USA). The reaction was incubated for 10 min at 25 °C, 60 min at 37 °C, 15 min at 70 °C, and 5 min at 4 °C. Afterward, cDNA was used as a template for amplification in real-time PCR reactions. Amplification was carried out in a 10-μL final volume containing 2 μL of cDNA, 1× Universal PCR Master Mix, and 1× specific Taqman primer/probe (Applied Biosystems, Foster City, CA, USA) for enlisted genes in Table S1 according to the manufacturer’s directions using a LigthCycler 96 instrument (Roche Molecular Systems, Pleasanton, CA, USA). The messenger RNA (mRNA) expression mean of duplicates per sample was normalized to 18S. Data were analyzed using the 2-Δct method [34].

### 2.13. Statistical Analysis

Data are presented as the mean ± SD or standard error of the mean (SEM). Cell culture data were obtained of independent triplicates for each experimental in vivo results from the number (*n*) of animals used. A Shapiro–Wilk test was used to establish normality of variables. Statistical significance was determined for parametric data with one-way ANOVA and a Tukey’s post-hoc test, Kruskal–Wallis and Mann–Whitney U test for non-parametric data, and a Fisher’s exact test for qualitative data (IBM SPSS Statistics V21.0; Armonk, NY, USA). A *p*-value <0.05 was considered as statistically significant.

## 3. Results

### 3.1. Dp Dose Selection in HepG2 Cells

**Figure 1:**
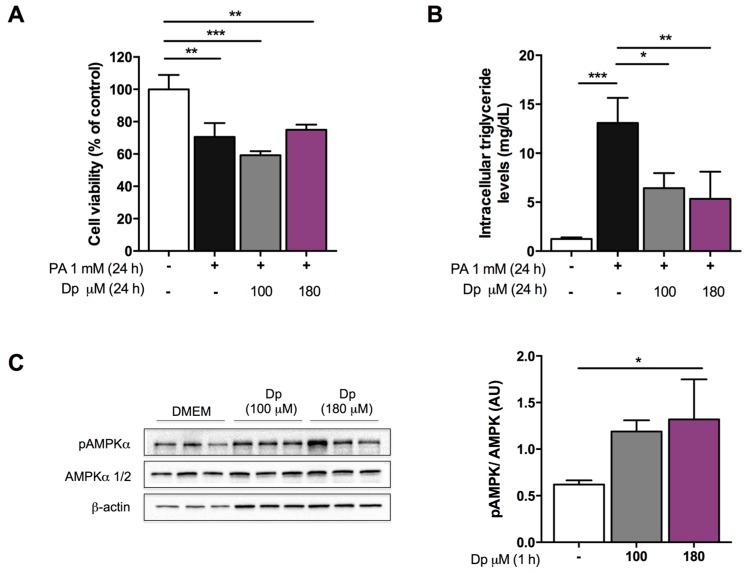
Delphinidin (Dp) dose selection based on its effects in human HepG2 cells. (**A**) Cell viability; (**B**) Total intracellular triglycerides; (**C**) AMP-activated protein kinase (AMPK) activation. In (**A**,**B**), cells were treated with Dp (100 μM and 180 μM) concomitantly with sodium palmitate (PA) for 24 h. In (**C**), cells were incubated with both Dp concentrations without PA for 1 h. One-way ANOVA and a Tukey’s post-hoc test were used for analysis; data represent the mean ± SD. * *p* < 0.05; ** *p* < 0.01; *** *p* < 0.001.

In order to establish an experimental Dp dose for consecutive experiments, the effects of Dp on cell viability, intracellular triglyceride accumulation, and AMPK phosphorylation levels were evaluated. The MTT assay showed that treatment with both Dp concentrations (100 μM and 180 μM) did not potentiate the cytotoxic effects caused by PA exposure for 24 h (Figure 1A). Triglyceride accumulation induced by exposure to PA for 24 h (13.09 ± 2.57 mg/dL) was significantly attenuated by Dp at the two concentrations tested (50% with 100 μM, and 59% with 180 μM). Similar triglyceride levels were observed with both Dp concentrations (6.42 ± 1.54 and 5.34 ± 2.77 mg/dL, respectively; Figure 1B).

Afterward, given the rapid degradation and limited bioavailability of Dp under cell culture medium [35], cells were Dp-treated without PA for 1 h. The results of immunoblotting showed that both concentrations effectively increased AMPK phosphorylation compared to DMEM-incubated cells considered as the control, although this effect was statistically significant only at the highest dose of Dp (Figure 1C). Therefore, a 180 μM Dp dose was selected to further investigate the molecular mechanisms involved in lipid metabolism that might have a key role diminishing intracellular triacylglycerol levels.

### 3.2. Effects of Dp on Lipid Metabolism of the Fatty Liver Cell Model

**Figure 2:**
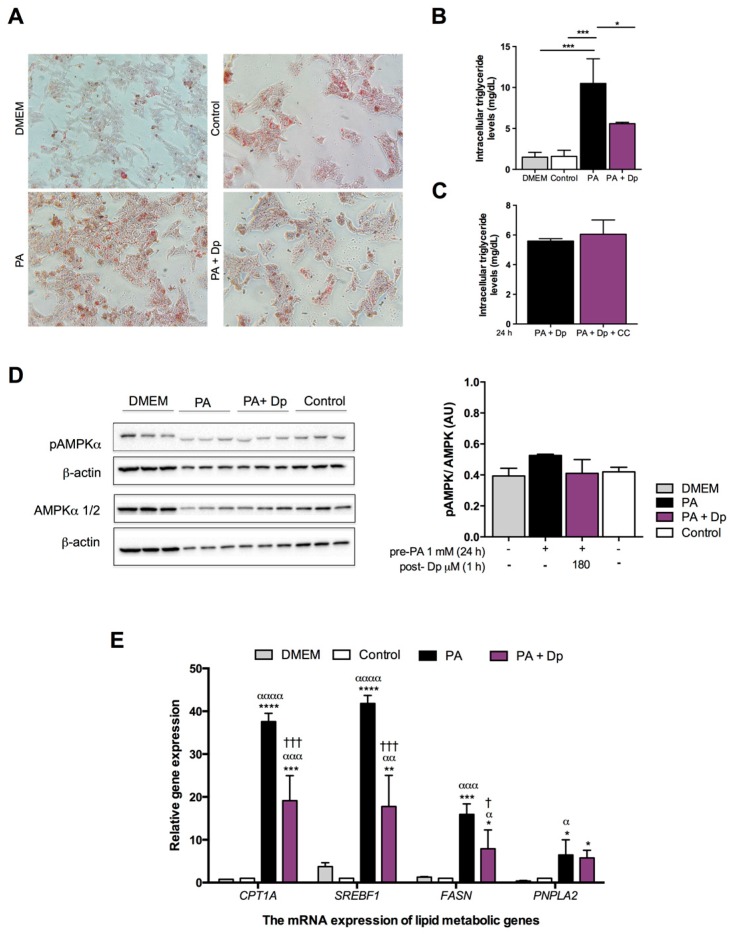
Protective effects of Dp on lipid metabolism against PA-induced lipid accumulation in HepG2 cells. (**A**) Representative photomicrographs of lipid droplets stained with Oil Red O (ORO) at 32× magnification; (**B**) Total intracellular triglycerides in HepG2 cells treated with 180 μM Dp and PA for 24 h; (**C**) Total intracellular triglycerides in HepG2 cells treated with 180 μM Dp, 20 μM compound C (CC), and PA for 24 h; (**D**) AMPK activation; (**E**) Gene expression of lipid metabolic genes in HepG2 cells pre-treated with PA for 24 h, and then additionally exposed to 180 μM Dp for 1 h. One-way ANOVA and a Tukey’s post-hoc test were used for analysis. In (**B**), data were analyzed with Kruskal–Wallis and Mann–Whitney U tests. The control was dimethyl sulfoxide + non-esterified fatty acid-free bovine serum albumin (DMSO + NEFA-free BSA). Data represent the mean ± SD. * *p* < 0.05; ** *p* < 0.01; *** *p* < 0.001; **** *p* < 0.0001. * vs. Dulbecco’s modified Eagle’s medium (DMEM); α vs. control; † PA vs. PA + Dp.

Dp treatment for 24 h displayed a significant reduction of lipid accumulation in cells exposed to PA, decreasing intracellular triglycerides levels by 47% (10.50 ± 3.00 vs. 5.59 ± 0.16 mg/dL; Figure 2A,B). Moreover, simultaneous cell incubation with CC did not attenuate the inhibitory effect of Dp in triglyceride overload raised by PA (5.59 ± 0.16 vs. 6.06 ± 0.96 mg/dL in the PA + Dp and PA + Dp + CC groups, respectively; Figure 2C). Interestingly, cells pre-treated with PA for 24 h and then exposed to Dp for 1 h did not display significant differences in AMPK phosphorylation levels among experimental groups (Figure 2D). Gene expression analysis was performed for genes involved in lipid metabolism: *CPT1A* (encoding CPT1A), *SREBF1* (encoding SREBP1c), *FASN* (encoding FAS), and *PNPLA2* (encoding ATGL). PA incubation induced a significant elevation in gene expression for all genes studied. The short Dp exposure for 1 h lowered mRNA levels for *CPT1A*, *SREBF1*, and *FASN*; noteworthy, these remained significantly different compared to cells incubated with DMEM or vehicles as the control (Figure 2E). No differences were observed for mRNA levels of *PNPLA2* between cells treated only with PA or PA supplemented with Dp.

### 3.3. Effects of Dp on the Body Weight, Liver Weight, Energy Intake, and Food Intake of Experimental Animals

**Figure 3:**
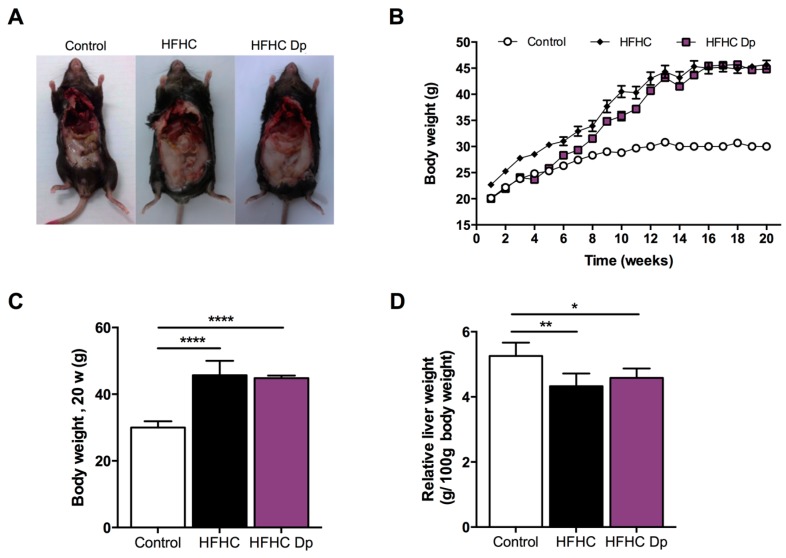
Effects of Dp on body and liver weight in high-fat/high-carbohydrate (HFHC)-diet-induced obese mice. (**A**) Representative images of gross contour of the whole body; (**B**) Body weight during experimental phases: prior to Dp treatment (1–16 weeks), and during Dp treatment (17–20 weeks). (**C**) Body weight at 20 weeks; (**D**) Relative ratio of fresh liver weight to body weight. One-way ANOVA and a Tukey’s post-hoc test were used for analysis. Data represent the mean ± SD. In (**B**), data represent the mean ± standard error of the mean (SEM). * *p* < 0.05; ** *p* < 0.01; **** *p* < 0.0001.

All animal groups gradually increased in body weight during the experimental period. Upon feeding on an HFHC diet for 16 weeks, mice significantly gained weight compared to the control (an increment of 50% in the HFHC group, and 51.3% in the HFHC-Dp group). At the end of the Dp treatment at 20 weeks, body weight did not significantly differ between the subgroups of HFHC and HFHC-Dp mice (45.67 ± 4.37 and 44.83 ± 0.75 g, respectively; Figure 3A–C). The ratio of relative fresh liver weight to body weight of the HFHC-fed mice was lower compared to the control, and no significant differences were observed after Dp administration (5.26 ± 0.29 vs. 4.33 ± 0.39 and 4.58 ± 0.29, control vs. HFHC and HFHC-Dp, respectively; Figure 3D).

As shown in Table 1, daily energy intake was comparable across all groups during the study (~11.23 ± 0.31 kcal/day); calories from dietary intake (food plus beverage) were taken into account to calculate the total daily energy intake. After 4 weeks of daily gavage, food-intake values of each group remained unaltered between experimental phases. The food intake was higher in the control group in both phases compared with the HFHC-fed mice groups, while the daily energy intake from fat was lower in the control group due to the fat energy percentage of the experimental standard diet. The energy intake from drinking water was similar in the HFHC-fed mice groups during the study phases, and was significantly different compared to the control.

### 3.4. Effects of Dp on Glucose Homeostasis and Insulin Sensitivity of Experimental Animals

**Figure 4:**
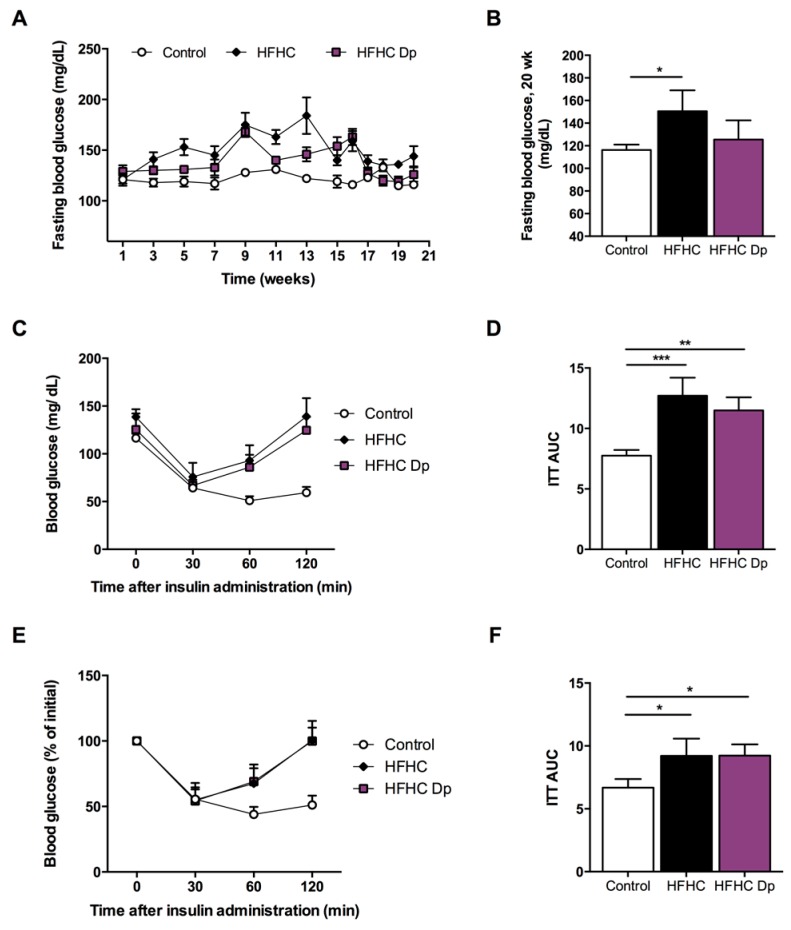
Effects of Dp on glucose homeostasis and insulin sensitivity in HFHC-diet-induced obese mice. (**A**) Fasting blood glucose levels during experimental phases: prior to Dp treatment (1–16 weeks), and during Dp treatment (17–20 weeks); (**B**) Fasting blood glucose levels at 20 weeks; (**C**,**D**) Fasting blood glucose levels during the insulin tolerance test (ITT), and the area under the curve (AUC); (**E**,**F**) Fasting blood glucose levels vs. initial values during the ITT, and the AUC. Control group, *n* = 3. One-way ANOVA and a Tukey’s post-hoc test were used for analysis. Data represent the mean ± SD. In (**A**), data represent the mean ± SEM. * *p* < 0.05; ** *p* < 0.01; *** *p* < 0.001.

Figure 4A shows the time-course blood-glucose concentration changes during the study for all experimental groups. The HFHC diet effectively increased glucose levels at the end of 16 weeks (116 ± 8 vs. 159 ± 26 and 163 ± 12 mg/dL in the control vs. HFHC and HFHC-Dp groups, respectively). After Dp treatment at 20 weeks, glucose levels were lower compared to the HFHC mice, although the observed reduction was not statistically significant (151 ± 18 vs. 121 ± 17 mg/dL in the HFHC and HFHC-Dp groups, respectively; Figure 4B). As expected, HFHC-fed mice groups showed an increase in the AUC during the ITT. The AUC values did not differ between the HFHC and HFHC-Dp mice (Figure 4C–F).

### 3.5. Effects of Dp on Lipid Metabolic Gene Expression of Experimental Animals

**Figure 5:**
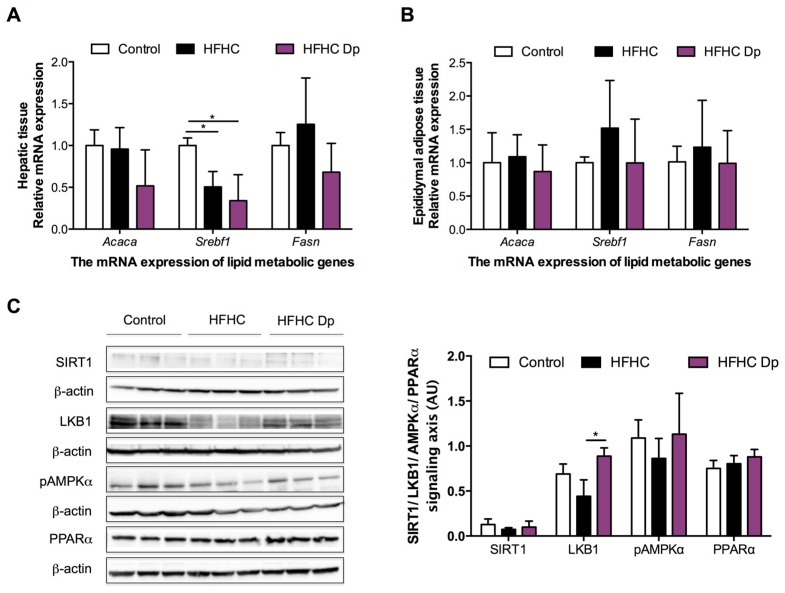
Effects of Dp on lipid metabolic gene expression, and sirtuin 1 (SIRT1)/liver kinase B1 (LKB1)/AMPK, and proliferator-activated receptor alpha (PPARα) signaling axis in HFHC-diet-induced obese mice. (**A**) *Acaca*, *Srebf1*, and *Fasn* messenger RNA (mRNA) expression in hepatic tissue; (**B**) *Acaca*, *Srebf1*, and *Fasn* mRNA expression in epididymal adipose tissue; (**C**) Relative protein levels of SIRT1, LKB1, phosphorylated AMPK (p-AMPKα), and PPARα in hepatic tissue. One-way ANOVA and a Tukey’s post-hoc test were used for analysis. Data represent the mean ± SD. * *p* < 0.05.

Gene expression was performed for genes involved in lipid synthesis: *Acaca* (encoding ACC1), *Srebf1* (encoding SREBP1c), and *Fasn* (encoding FAS) in both hepatic and epididymal adipose tissue. Hepatic *Srebf1* mRNA levels in HFHC-fed mice groups were lower than in the control group, and no differences between HFHC and HFHC-Dp groups were observed (Figure 5A). Dp treatment reduced hepatic *Fasn* levels; however, it was not statistically significant. The *Acaca* expression did not change after exposure to an HFHC diet or Dp. The *Acaca*, *Srebf1,* and *Fasn* mRNA levels of epididymal adipose tissue remained unaffected by the HFHC diet and the Dp treatment (Figure 5B).

### 3.6. Effects of Dp on the SIRT1/LKB1/AMPK and PPARα Signaling Axis of Experimental Animals

The HFHC exposure elicited a reduction in LKB1 and p-AMPK protein levels; however, this was not statistically significant. Dp treatment reverted the HFHC effects on LKB1 and p-AMPK (the latter did not reach significance). Nevertheless, global immunoblotting analysis revealed no changes in the SIRT1/LKB1/AMPK and PPARα pathway among the experimental groups (Figure 5C).

### 3.7. Effect of Dp on Liver Histology

**Figure 6:**
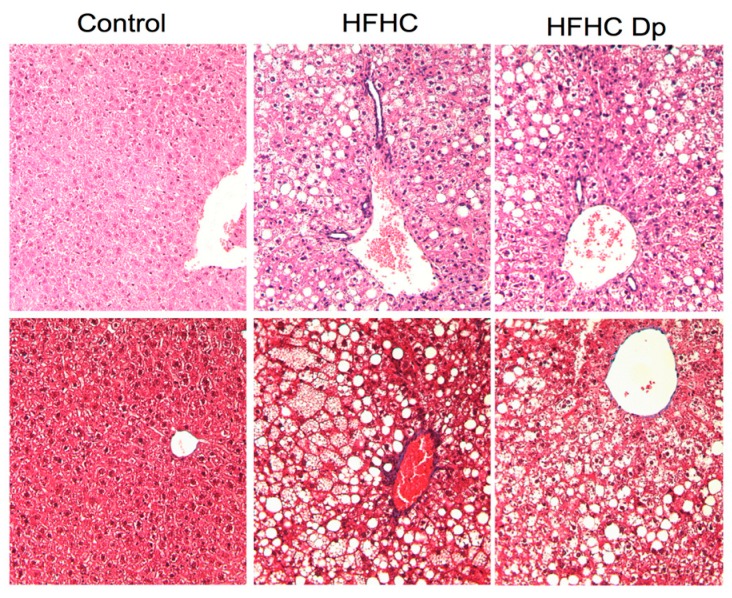
Effects of Dp on the liver histology of HFHC-diet-induced obese mice. Representative photomicrographs after hematoxylin and eosin (H&E; upper panels) and Masson’s trichrome (lower panels) staining at 20× magnification.

Histological examination revealed significant severe hepatic damage in both HFHC groups. No significant changes were observed in the degree of steatosis, hepatocyte ballooning, and lobular inflammation with Dp treatment; thus, the NAS score was similar for both groups. The extracellular matrix and portal inflammation findings were comparable across experimental groups (Figure 6; Table 2).

## 4. Discussion

In the present study, we examined, for the first time, the effects of Dp on metabolic alterations associated with NAFLD in HepG2 cells and HFHC-diet-induced obese mice. Additionally, we explored possible molecular mechanisms underlying the Dp effects on lipid metabolism. Our results showed that Dp effectively reduced intracellular triglyceride accumulation in HepG2 cells by downregulating the gene expressions of *CPT1A*, *SREBF1*, and *FASN* without modifying AMPK activity. In light of our in vitro findings, the effects of Dp were subsequently investigated in an intervention study in vivo.

In agreement with an earlier published study, the PA-induced fatty liver cell model provoked increased lipid intracellular accumulation and upregulated mRNA levels of *FASN*, *CPT1A*, and *SREBF1* genes in hepatocytes [36]. Here, the triglyceride accumulation was substantially reduced by ~53% with Dp treatment. A comparable inhibitory effect on triglyceride accumulation induced by oleic acid in HepG2 was observed with anthocyanin-rich extracts from blackberry, wild blueberry, strawberry, and chokeberry. Furthermore, the effectiveness of purified Dp-3-glucoside (86 μM) in inhibiting triglyceride accumulation was approximately 35% [33]. A similar effect was found with cyanidin treatment (100 μM) in HepG2 cells incubated with a mixture of fatty acids where triglyceride overload was reduced by 23% [29]. Previous studies showed that AMPK activation by anthocyanins underlies lipid metabolism regulation [25]; however, our data in HepG2 cells suggest that Dp may provoke beneficial effects in the prevention of triglyceride accumulation by downregulating the gene expressions of the SREBP1c, FAS, and CPT1A proteins without modifying AMPK activity.

As expected, chronic HFHC exposure induced increased adiposity, insulin resistance, and severe hepatic damage characterized by steatosis and inflammation in mice. Interestingly, mice on an HFHC diet consumed less food than those on a standard diet. Despite this reduction, the energy intake remained similar among groups due to the increase in the proportion of calories from fat in the HFHC diet, as well as the energy from the drinking water. In line with our results, other studies reported similar dietetic patterns, as well as their effect on body weight [37,38,39]. C57BL/6J mice on an HFD for 15 weeks gained weight in a comparable way (~44 g of body weight) to that observed herein despite food consumption being lower, and the energy intake was not significantly different compared to those fed with a low-fat diet [38]. Similarly, after 12 weeks on a high-fat/ high-calorie diet (45% kcal from fat), but not on a high-complex-carbohydrate/high-calorie diet (HCD; 70% kcal from carbohydrates), mice showed increased body weight and fat mass (%). This effect on body composition was observed despite both HFD- and HCD-fed mice consuming a minor amount of food compared to the control group, and the calorie intake was not different among experimental groups [39]. Those findings suggest that the increment in dietary fat generates distinct metabolic responses.

Among mouse strains, the inbred strain C57BL/6 displays a genetic predisposition to gaining weight on an HFD. In a previous published study, it was described that there exists an interaction between dietary fat content and body weight. The effects of four different diets on body composition in two different mouse strains were tested: high-fat/high-sucrose (HH; 5.55 kcal/g), high-fat/low-sucrose (HL; 5.55 kcal/g), low-fat/high-sucrose (LH; 4.07 kcal/g), and low-fat/ low-sucrose (LL; 4.07 kcal/g). It was observed that both A/J and C57BL/6 strains gained more body and fat-pad weight when they were fed an HFD (58% kcal from fat) for 16 weeks, though the increase was greater in B6 mice (31.8 and 46.8 g of body weight in A/J and B6 mice, respectively). Final body weights reported are comparable to those observed in the present study after 16 weeks on the experimental diet. Furthermore, both A/J and B6 strains gained fat mass; however, the effects of the HFD were much greater in B6 mice (32.2 and 37.2% in A/J and B6 mice, respectively), and it was clearly reduced when they were fed with an LH diet. Furthermore, B6 mice exposed to high fat content have a higher feed efficiency (grams gained per kcal consumed) than the A/J strain. In other words, B6 mice gain more weight for each calorie consumed on HFD feeding. As a conclusion, beyond the caloric density, the response to certain dietary compositions is determined by genetic factors of each strain of mouse. In this case, a high proportion of calories from fat provoked changes in the body composition of a B6 strain, increasing fat mass [40]. In addition to the genetic susceptibility of C57BL/6 to developing obesity, it was demonstrated in other animal models that not only does the caloric intake play a pivotal role in the development of metabolic diseases associated with obesity due to each macronutrient generating different biological responses, but also the macronutrient distribution [41].

Hepatic lipid accumulation can be originated from an increase in de novo lipogenesis due to a deregulation of SREBP1c activity, though ectopic fat in the liver is due, in great measure, to an elevated lipid influx from hypertrophied insulin-resistant adipose tissue [42]. In humans, the hepatic expression of *SREBF1*, as well as its target lipogenic genes, varies according to the severity of fatty liver. SREBP1c increases in SS, whereas NASH is associated with a reduction in both the protein and mRNA levels of SREBP1c [43], which correlates with our results in the animal model. We observed a significant reduction in the hepatic gene expression of *Srebf1* after an HFHC diet in mice classified with NASH by the NAS. AMPK represses anabolic processes such as fatty-acid synthesis by directly inhibiting SREBP1c. It was reported that chronic HFD (60% kcal from fat) exposure reduced AMPK activity by approximately 15% in rats, though the gene expression of catalytic subunits did not change [44]. Nevertheless, consistent with a previous report, we did not observe changes in hepatic p-AMPK protein levels here after experimental feeding [45]. Nonetheless, the role of AMPK in the development of diet-induced fatty liver involves the reduction of hepatic triacylglycerol in a LKB1-dependent manner due to AMPK reactivation [46].

Isolated anthocyanins and anthocyanin-rich extracts exert beneficial metabolic effects. Anthocyanins from purple sweet potato improved the glycemic index, reduced weight gain, and attenuated diet-induced (45% kcal from fat) hepatic lipid accumulation via AMPK-mediated modulation of fatty-acid metabolism [25]. Likewise, isolated Dp-3-sambubioside-5-glucoside from maqui berry improved fasting glycemic indices in diet-induced obese mice [47]. Nevertheless, the precise effect of the aglycone Dp on metabolic alterations associated with NAFLD in an obese mice model is yet to be reported. In the present study, Dp administration did not display benefits regarding any of the studied parameters. In line with this, no effects on molecular mechanisms were observed. To our best knowledge, this is the first study investigating the hepatic effects of Dp on the SIRT1/LKB1/AMPK and PPARα signaling axis in an HFHC-diet-induced obesity model of NAFLD. Despite an increase in relative levels of LKB1 after Dp administration, the global immunoblotting analysis revealed no significant changes in the pathway mentioned, as well as in the mRNA expression of lipogenic genes, such as *Acaca* and *Fasn*, in hepatic tissue, or *Srebf1*, *Acaca*, and *Fasn* in epididymal adipose tissue, due to the HFHC diet or Dp treatment. Our findings support a previous report, in which an anthocyanin-rich bilberry extract administrated for a long period (24 weeks) in mice did not prevent either visceral fat-mass gain or hepatic lipid accumulation (triglycerides and total cholesterol) raised by an HFD (45% kcal from fat) [48].

Discordance between our results and previous studies showing beneficial effects on metabolic alterations could be potentially explained by differences in the methodological approach. In the first place, anthocyanin doses were higher in other experimental reports [47,49]. Here, we calculated Dp dose based on the total intake of anthocyanins consumed daily by humans. The estimated daily intake of total anthocyanins obtained from the National Health and Nutrition Examination Survey was 12.5 mg/day in a typical United States of America (USA) diet [50], while European anthocyanin consumption varies between 19.8 and 64.9 mg/day according to each country [13]. Therefore, we tested a Dp dose that could easily be reached by humans with a regular diet: 15 mg/kg bw/day of Dp corresponding to a 70-kg human consuming 85 mg of total anthocyanins (~25 g of fresh blueberries, *Vaccinium corymbosum*) based on body surface normalization [11,51]. Similar Dp doses were effective in other disease models [52,53]. A previous study using Dp-3-sambubioside-5-glucoside from maqui berry (*Aristotelia chilensis*) at doses of 425 mg/kg bw/day, a dose approximately 28-fold higher than ours, reported anti-diabetic effects [47]. Since anthocyanins have limited bioavailability [54], increased plasma concentrations by higher doses may promote health benefits. Nevertheless, Dp-3-*O*-glucoside at a similarly high oral dose (300 mg/kg bw/day) did not affect hyperglycemia while maldivin-3-*O*-glucoside lowered glucose levels by 34%. [49]. These data suggest that, in addition to dose sizes, the specific chemical structure of anthocyanins, determined by the type of aglycones or sugar moieties, also influence their bioavailability and biological properties. In this regard, time-course plasma concentrations after an oral administration of Dp-3-rutinoside were lower compared to those seen with cyanidin-3-rutinoside, and concentrations after the latter were higher than those seen with cyanidin-3-glucoside after 30 minutes of administration in rats [55]. In humans, changes in plasma concentrations of Dp-based anthocyanins varied according to the nature of the sugar, whereby the decline in Dp-3-rutinoside levels was gentler than those of Dp-3-glucoside [55]. Considering these findings together, differences between our results and the previously reported beneficial effects on metabolic alterations mostly with cyanidin-based anthocyanins could be explained. Nonetheless, it was reported that only anthocyanin extracts from Moro orange juice, but not purified cyanidin-3-glucoside (90 mg/kg bw/day), reduced fat-mass accumulation [56]. It suggests that other compounds such as another polyphenols present in foods, as well as extracts, might potentiate the anthocyanin effects, instead of a single anthocyanin being responsible for health benefits. A recent study showed that cyanidin-3-glucoside (200 mg/kg bw/day) improved steatosis, in which the levels of hepatic triglycerides and total cholesterol were reduced after eight weeks of treatment. Although triglyceride levels were lower after anthocyanin administration in this study, and since hepatic triglyceride accumulation is the hallmark of NAFLD, it is questionable that these levels were not significantly higher after a high-fat/high-cholesterol diet (17% fat (*w*/*w*) in addition to high cholesterol) in the NAFLD model [57]. Hence, a discrepancy among experimental diets used to induce fatty liver could influence outcomes.

In contrast with other studies, we used a more aggressive diet to induce obesity-related NAFLD. We provided a high-fat diet in addition to high-carbohydrate drinking water, known as the Western-style diet, while most of the previous studies with anthocyanins induced NAFLD only via excessive fat feeding (45–60% kcal from fat) for a less prolonged duration (8–12 weeks) [23,25]. Our experimental drinking water contained 4.2% of sugars with a high fructose proportion. It was demonstrated that the consumption of an HFD supplemented with a 30% fructose-rich beverage for 10 weeks provoked increases in weight, liver weight, visceral fat, fasting blood glucose levels, and insulin resistance levels compared with those that only consumed an HFD. In the same study, steatosis was graded as moderate in mice fed with an HFD alone; in contrast, fructose supplementation produced severe hepatic steatosis [58]. Additionally, the progression of NAFLD was quicker with a Western-style diet [59]. In the present study, instead of just evaluating the effects on hepatic lipid content, the Dp effects on hepatic damage caused by a chronic Western-style diet were also evaluated using the NAS. Severe steatosis accompanied by lobular inflammation with ballooning was not improved with Dp treatment. Furthermore, most studies showing improvements on metabolic alterations related to NAFLD by anthocyanins were carried out with a preventive approach, namely anthocyanin treatment started at the same time as the experimental diet [21,23,24]. Here, we investigated the potential of Dp as a short-time intervention after the establishment of diet-induced NAFLD, as well as other associated chronic metabolic features. Collectively, the previously mentioned methodological arguments potentially limited the expected Dp effects in mice that we observed in vitro, as well as in other previous investigations with anthocyanins.

In summary, we found that Dp effectively reduced intracellular lipid accumulation in vitro through the modulation of the gene expressions of lipid metabolic genes. Notwithstanding, when we extrapolated our investigation to an animal model that simulated, more realistically, the chronic dietary patterns of obese patients with NAFLD, as well as the severe metabolic disturbances, we did not observe any metabolic amelioration due to short Dp intervention. Possibly, the role of Dp, as well as other anthocyanins, on metabolic disorders of NAFLD, is to counteract instead of reversing chronic damage. Therefore, further studies are needed to elucidate their metabolic effects, as well as the action mechanisms, in order to establish an anthocyanin dietary recommendation to counteract the harmful effects of a Western-style diet, or to help in the effective treatment of NAFLD.

## Figures and Tables

**Table 1:** Dietary ingestion during experimental phases.

	Control	HFHC	HFHC-Dp
**Prior to Dp treatment**			
Energy intake (kcal/day)	11.15 ± 0.08 ^a^	11.7 ± 0.59 ^a^	11.27 ± 0.16 ^a^
Food intake (g/day)	3.66 ± 0.03 ^a^	2.42 ± 0.13 ^b^	2.33 ± 0.04 ^b^
Energy intake from fat (kcal/day)	3.21 ± 0.02 ^a^	5. 02 ± 0.27 ^b^	4.83 ± 0.09 ^b^
Energy intake from fat (% kcal)	28.8	45	45
High-carbohydrate drinking water intake (mL/day)	0.00 ± 0.00 ^a^	3.27 ± 0.12 ^b^	3.22 ± 0.15 ^b^
Energy intake from high-carbohydrate drinking water (kcal/day)	0.00 ± 0.00 ^a^	0.55 ± 0.02 ^b^	0.54 ± 0.03 ^b^
**During Dp treatment**			
Energy intake (kcal/day)	10.74 ± 0.08 ^a^	11.21 ± 1.28 ^a^	11.19 ± 1.36 ^a^
Food intake (g/day)	3.53 ± 0.03 ^a^	2.33 ± 0.28 ^b^	2.33 ± 0.29 ^b^
Energy intake from fat (kcal/day)	3.09 ± 0.02 ^a^	4.81 ± 0.57 ^b^	4.82 ± 0.60 ^b^
Energy intake from fat (% kcal)	28.8	45	45
High-carbohydrate drinking water intake (mL/day)	0.00 ± 0.00 ^a^	3.06 ± 0.14 ^b^	2.93 ± 0.20 ^b^
Energy intake from high-carbohydrate drinking water (kcal/day)	0.00 ± 0.00 ^a^	0.51 ± 0.02 ^b^	0.49 ± 0.03 ^b^

Values are expressed as the mean ± SD. HFHC, high-fat diet plus high-carbohydrate drinking water; HFHC-Dp, high-fat diet and high-carbohydrate drinking water plus Dp at 15 mg/kg body weight/day. Kruskal–Wallis and Mann–Whitney U tests were used for analysis. The means marked with superscripted letters were significantly different compared with the other groups.

**Table 2:** Hepatic histological features.

	Grade	Control (*n* = 6)	HFHC (*n* = 6)	HFHC-Dp (*n* = 6)
Steatosis	0/1/2/3	6/0/0/0 ^a^	0/0/0/6 ^b^	0/0/1/5 ^b^
Lobular inflammation	0/1/2/3	6/0/0/0 ^a^	0/0/2/4 ^b^	0/0/0/6 ^b^
Ballooning	0/1/2	6/0/0/0 ^a^	0/3/3 ^b^	0/3/3 ^b^
NAS score *		0 ± 0.00 ^a^	7.17 ± 0.98 ^b^	7.33 ± 0.82 ^b^
Portal inflammation	0/1/2/3/4	6/0/0/0 ^a^	1/5/0/0 ^a^	1/5/0/0 ^a^
Fibrosis	0/1/2/3/4	6/0/0/0 ^a^	5/1/0/0 ^a^	6/0/0/0 ^a^

Liver damage was classified evaluating steatosis (grade 0–3), lobular inflammation (grade 0–3), ballooning (grade 0–2), portal inflammation (grade 0–4), and fibrosis (grade 0–4). Data represent the number of animals in the group at the given score; *p* < 0.05 calculated with a Fisher’s exact test. * The results of the non-alcoholic fatty liver disease (NAFLD) activity score (NAS) are represented as the mean ± SD; *p* <0.05 calculated with Kruskal–Wallis and Mann–Whitney U tests. Values marked with superscripted letters were significantly different compared with the other group (a vs. b).

## Supplementary Materials

The following are available online at http://www.mdpi.com/2072-6643/10/8/1060/s1. Figure S1: Effects of PA on intracellular lipid accumulation in HepG2 cells; Table S1: Specific Taqman primers/probe used to evaluate the gene expression of genes of interest.

## Abbreviations

ACC1	acetyl-CoA carboxylase 1
AMPK	AMP-activated protein kinase
ATGL	adipose triglyceride lipase
AUC	area under the curve
Bw	body weight
CC	compound C
cDNA	complementary DNA
CPT1A	carnitine palmitoyltransferase 1A
DMEM	Dulbecco’s modified Eagle’s medium
DMSO	dimethyl sulfoxide
dNTP	deoxynucleotide triphosphate
Dp	delphinidin
DTT	dithiothreitol
FAS	fatty acid synthase
FBS	fetal bovine serum
H&E	hematoxylin and eosin
HCD	high-complex-carbohydrate/high-calorie diet
HFD	high-fat diet
HFHC	high-fat and high-carbohydrate diet
HH	high-fat/high-sucrose diet
HL	high-fat/low-sucrose diet
ITT	insulin tolerance test
LH	low-fat/high-sucrose diet
LKB1	liver kinase B1
LL	low-fat/low-sucrose diet
M-MLV	Moloney Murine Leukemia Virus
mRNA	messenger RNA
MTT	3-(4,5-dimethylthiazol-2-yl)-2,5-diphenyltetrazolium bromide
NAFLD	non-alcoholic fatty liver disease
NAS	NASH activity score
NASH	non-alcoholic steatohepatitis
NEFA-free BSA	non-esterified fatty-acid-free bovine serum albumin
ORO	Oil Red O staining
PA	sodium palmitate
PBS	phosphate-buffered saline
PVDF	polyvinylidene difluoride
SEM	standard error of the mean
PPARα	peroxisome proliferator-activated receptor alpha
SIRT1	sirtuin 1
SREBP1c	sterol regulatory element binding protein-1c
SS	simple steatosis
