# Supplementary material for: Delphinidin Ameliorates Hepatic Triglyceride Accumulation in Human HepG2 Cells, but Not in Diet-Induced Obese Mice
Source: Nutrients. 2018 Aug 10;10(8):1060. doi: 10.3390/nu10081060 (PMC6115893; doi:10.3390/nu10081060)
Supplement: Supplementary file 1 [file nutrients-10-01060-s001.pdf]

Supplementary materials

Effect of PA on intracellular lipid accumulation in HepG2 cells

Cells treated with both 0.5 mM and 1 mM sodium palmitate (PA) concentrations for 24 h increased total intracellular lipid accumulation visualized by ORO staining (Figure S1A), and no significant differences were observed between both PA concentrations evaluated by the pixel density of 20 images per group (Figure S1B). Furthermore, PA elevated triglycerides in a dose-dependent manner compared to BSA-treated cells considered as control (1.25 ± 0.15 vs 6.75 ± 1.26 and 15.74 ± 3.38 mg/dL, respectively) (Figure S1C). Based on the results, the 1 mM PA concentration was selected for further experiments.

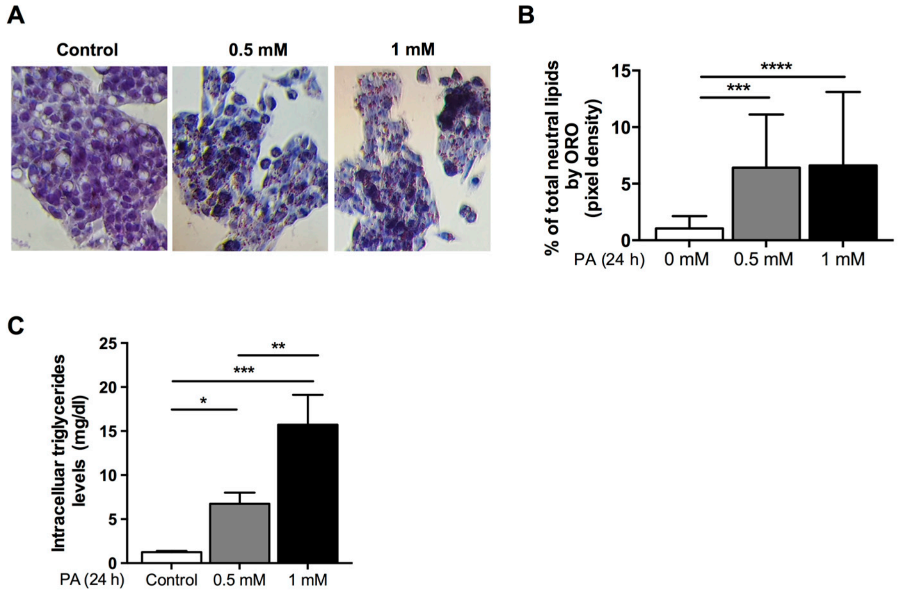

Figure S1. Effects of PA on intracellular lipid accumulation in HepG2 cells. (**A**) Total lipid accumulation visualized by H&E and ORO staining at 32× magnification, (**B**) percentage of total neutral lipids stained by ORO, and (**C**) total intracellular triglycerides. One-way ANOVA and Tukey's post-hoc test. In B, values are the mean of 20 random images per each group. Data represent mean ± SD. (* $p < 0.05$, ** $p < 0.01$ *** $p < 0.001$)

Table S1. Specific Taqman primers/probe used to evaluate the gene expression of genes of interest.

Gene	Protein	Specie	Taqman Probe	Accession Number
SREBF1	SREBP1c	Human	Hs01088679_g1	NM_001005291
Srebf1		Mouse	Mm00550338_m1	NM_011480
FASN	FAS	Human	Hs01005622_m1	NM_004104
Fasn		Mouse	Mm00662319	NM_007988
PNPLA2	ATGL	Human	Hs00386101_m1	NM_020376
CPT1A	CPT1A	Human	Hs00912671_m1	NM_001031847
Acaca	ACC1	Mouse	Mm01304257_m1	NM_133360
